# Characteristics of *Helicobacter pylori* Heteroresistance in Gastric Biopsies and Its Clinical Relevance

**DOI:** 10.3389/fcimb.2021.819506

**Published:** 2022-02-04

**Authors:** You-hua Wang, Xiao-ling Gong, Ding-wei Liu, Rong Zeng, Lin-fu Zhou, Xiao-yan Sun, Dong-sheng Liu, Yong Xie

**Affiliations:** ^1^Department of Gastroenterology, The First Affiliated Hospital of Nanchang University, Nanchang, China; ^2^Department of Blood Transfusion, The First Affiliated Hospital of Nanchang University, Nanchang, China; ^3^Department of Biochemistry, Department of the Children’s Hospital, National Clinical Research Center for Child Health, Zhejiang University School of Medicine, Hangzhou, China; ^4^Department of Epidemiology, Bethune International Peace Hospital, Shijiazhuang, China

**Keywords:** *Helicobacter pylori*, heteroresistance, clarithromycin, levofloxacin, peptic ulcer

## Abstract

**Background:**

Antimicrobial susceptibility testing (AST) plays a vital role in anti-*Helicobacter pylori* treatment, but the traditional AST method has difficulty detecting heteroresistance, which may cause an increased prevalence of resistant strains and eradication failure.

**Aims:**

To investigate the characteristics of heteroresistance in *H. pylori* in gastric biopsies and investigate its clinical relevance.

**Method:**

A total of 704 gastric biopsies were selected for 23S rRNA and gyrA gene sequencing, 470 *H. pylori* isolates from these biopsies were selected for AST, and the clinical characteristics of the patients were reviewed.

**Result:**

For the 699 biopsies that were positive for 23S rRNA gene, 98 (14.0%) showed a heteroresistance genotype, and a wild type (WT) combined with A2143G (86.7%) genotype was found in most samples. For the 694 biopsies that were positive for gyrA gene, 99 (14.3%) showed a heteroresistance genotype, and a WT combined with 87K (26.3%) or WT combined with 91N (23.2%) genotype was predominant. According to the E-test results, the resistance rates of heteroresistance genotype samples for clarithromycin and levofloxacin were 36.2% and 68.1%, respectively. When dividing the heteroresistance samples into different groups according to the sequencing profile peaks of the mutation position, the resistance rates were higher along with mutation peaks at the mutation position. In addition, patients infected with mutated or heteroresistant strains showed lower peptic ulcer detection rates than those infected with the WT strain (p < 0.05).

**Conclusion:**

Heteroresistance genotypes for clarithromycin and levofloxacin were not rare in *H. pylori*. Most cases with a heteroresistance genotype showed a susceptible phenotype for clarithromycin and a resistance phenotype for levofloxacin. Patients infected with heteroresistance genotype strains showed a lower peptic ulcer detection rate than those infected with the WT strain.

## Introduction

*Helicobacter pylori* is a gram-negative, helix-shaped, microaerophilic bacterium that colonizes the human stomach. *H. pylori* infection can last for decades and lead to gastrointestinal diseases such as peptic ulcer disease, chronic gastritis, and gastric adenocarcinoma (Wroblewski and Peek, 2016; Narayanan et al., 2018). Therefore, the eradication of *H. pylori* infection has been widely recommended by several consensus opinions or guidelines (Sugano et al., 2015; Malfertheiner et al., 2017). Currently, the main strategy for *H. pylori* eradication is an empirical treatment based on local antimicrobial susceptibility data or tailored treatment based on individual antimicrobial susceptibility testing (AST) (Flores-Trevino et al., 2018). This means that choosing antimicrobials to which the pathogen is susceptible is important for anti-*H. pylori* treatment (Savoldi et al., 2018). Our previous work proved that antibiotic resistance causes a significant decrease in the eradication rate of *H. pylori*, especially for clarithromycin (CLA)-based treatment and levofloxacin (LEV)-based treatment (Zou et al., 2020).

AST plays a vital role in anti-*H. pylori* treatment, and traditional methods, including agar dilution experiments, disk diffusion tests, and epsilometer tests (E-tests), define a susceptible phenotype or resistance phenotype, but there is still a third status named heteroresistance. It refers to a phenomenon where there are different subpopulations of seemingly isogenic bacteria that exhibit a range of susceptibilities to a particular antibiotic (El-Halfawy and Valvano, 2015; Andersson et al., 2019). Heteroresistance has been reported to a wide variety of antibiotics and is common in several bacterial species. Jo et al. showed that among tigecycline-susceptible and intermediate-resistant *Acinetobacter baumannii* isolates, 56.2% and 59.5% isolates were identified as heteroresistant to tigecycline, respectively (Jo and Ko, 2021). Jia et al. found a high prevalence (57.3%) of heteroresistance to cefepime in *Pseudomonas aeruginosa* (Jia et al., 2020). Our previous work showed that the frequencies of CLA and LEV heteroresistance in *H. pylori* strains were approximately 18% and 20%, respectively (Wang et al., 2020).

Heteroresistance cases are composed of a resistant subpopulation of cells and a susceptible population; some would regard susceptible populations as the majority population, which may lead to a susceptible phenotype, while the susceptible data for heteroresistance cases were currently limited. Besides, bacteria resistance to the antibiotic may also be varied. For example, a resistance genotype of CLA could be mutated as A2142C/G or A2143G in 23S rRNA gene, and resistance genotype of LEV could be mutated in the 87th amino acid and/or 91st amino acid (Wang et al., 2020; Domanovich-Asor et al., 2021; Kim et al., 2021). Does heteroresistance for these antibiotics show different characteristics?

How would heteroresistance cases affect clinical outcomes? Some research would regard heteroresistance as a point along the evolutionary path, and this may often develop before resistance and frequently be a stage in its progression (Band and Weiss, 2021). And the clinical significance of heteroresistance has been the subject of debate; many studies, such as *in vitro* experiments, mathematical modeling, animal infection models, and clinical studies, have shown that resistant subpopulations can be enriched during antibiotic exposure and might eventually lead to treatment failure (Kao et al., 2014; Band and Weiss, 2019; Zhang et al., 2021). Some studies have proven that virulence factors, such as vacA, babA2, and oipA, are related to an increased risk of peptic ulcer disease in subjects with *H. pylori* infection (Kishk et al., 2021; Shahini et al., 2021). Do patients infected with heteroresistance genotype strains have different clinical outcomes? To investigate these questions, we performed this study.

## Methods

### Patients and Biopsy

Outpatients referred for gastroscopy at the First Affiliated Hospital of Nanchang University between June 2018 and June 2021 and who were positive on a urea breath test or histopathology were enrolled. Patients would exclude if they received anti-*H. pylori* treatment previously. Written informed consent was obtained from each participating patient before enrollment in the study. After the biopsy was obtained, part of them were sent to pathology laboratory in our hospital for further analysis. The research protocol was approved by the Ethics Committee of the First Affiliated Hospital of Nanchang University (IRB 2018-116). Patients were excluded if they were taking a proton pump inhibitor (PPI) or H2 receptor antagonists within 4 weeks prior to enrollment.

### *Helicobacter pylori* Culture and E-Test Method

Briefly, gastric mucosal biopsy specimens from the antrum and corpus were mixed and stored in brain heart infusion broth (Oxoid, Basingstoke, UK) with 20% glycerin at −80°C before use. After homogenization, gastric mucosal biopsies were cultured on Campylobacter agar (Oxoid, Basingstoke, UK) plates supplemented with 5% defibrinated sheep blood (Bio-Kont, Zhejiang, China) supplemented with 2.5 mg/L of vancomycin, 3 mg/L of trimethoprim, 2 mg/L of polymyxin B, and 2 mg/L of amphotericin B (Duly Biotech, Nanjing, China). The plates were incubated in a microaerobic atmosphere (10% CO_2_, 5% O_2_, and 85% N_2_) at 37°C for up to 5 days.

### Antimicrobial Susceptibility Testing

Susceptibility to CLA and LEV was assessed using the E-test method. The resistance breakpoints for CLA and LEV were set at >0.5 and >1 mg/L, respectively, which were selected using breakpoint tables for interpretation of minimal inhibitory concentrations (MICs) provided by the European Committee on Antimicrobial Susceptibility Testing version 9.0, 2019 (http://www.eucast.org). *H. pylori* culture and antibiotic susceptibility testing were performed by the Institute of Gastroenterology and Hepatology of the First Affiliated Hospital of Nanchang University.

### Genomic DNA Extraction, Sequencing, and Mutation Analysis

DNA was extracted from biopsy samples using a QIAamp1 DNA Mini Kit (Qiagen, Hilden, Germany) according to the manufacturer’s instructions. PCR for 23S rRNA and gyrA was performed: 23S rRNA_F (5′-TAACAGAAACATCAAGGGTGGTA TC-3′), 23S rRNA_R (5′-CTATAACGGTCCTAAGGTAGCGA-3′) (product 281 bp), gyrA_F (5′-AAGTGGGGAT TGATTCTTCTATTGA-3′), and gyrA_R (5′-ATTTCTTCACTCGCCTTAGTCATTC-3′) (product 373 bp). The PCR product was purified with a DNA product purification kit (Shenggong Biological Engineering, Shanghai, China), and sequencing was performed with first-generation Sanger sequencing.

Sequence data were analyzed using DNAMAN software (2005, Lynnon), and heteroresistance status was evaluated with ContigExpress (2000, InforMax) and compared with a reference sequence (*H. pylori* 26695). Sequence data were examined in terms of codons for 23S rRNA, and comparisons were performed for the amino acids of gyrA gene. The resistance genotype for CLA was defined as A2142C, A2142G, and A2143G mutations in 23S rRNA gene (Wang et al., 2020; Anis et al., 2021). The wild-type (WT) genotype for CLA was defined as a negative mutation in the above site. The heteroresistant genotype for CLA was defined as having these two genotypes. The WT genotype for LEV was defined as the 87th asparagine and 91st aspartate in gyrA gene. The resistance genotype for LEV was defined as the 87th asparagine, and/or 91st aspartate was replaced by other amino acids (Binkowska et al., 2018). The heteroresistant genotype for LEV was defined as having these two genotypes.

We divided heteroresistance cases according to the sequencing profile peaks of the mutation position, and a difference of fewer than 1/3 times was defined as similarly high. When the mutation peak was significantly higher than the WT peak, this sample was designated the MUT>WT group; when the mutation peak was significantly lower than the WT peak, this sample was designated the MUT<WT group; and when the mutation peak was similar to the WT peak, this sample was designated the MUT≈WT group (**Supplementary Figure 1**).

### Statistical Analysis

SPSS Statistics for Windows (version 21.0, IBM Corp, Armonk, NY, USA) was used to perform all statistical analyses. Chi-square and Fisher’s exact tests were used to determine the statistical significance of differences between categorical variables. A p-value ≤0.05 was considered statistically significant.

## Results

Gastric biopsies from 704 patients were included in this study. Among these biopsies, 699 were positive for CLA resistance gene 23S rRNA detection. A total of 694 were positive for the LEV resistance gene gyrA, and 470 *H. pylori* isolates from these biopsies were selected to perform CLA and LEV MIC data for further analysis.

### Profile of Heteroresistance in Biopsies

**Figure 1:**
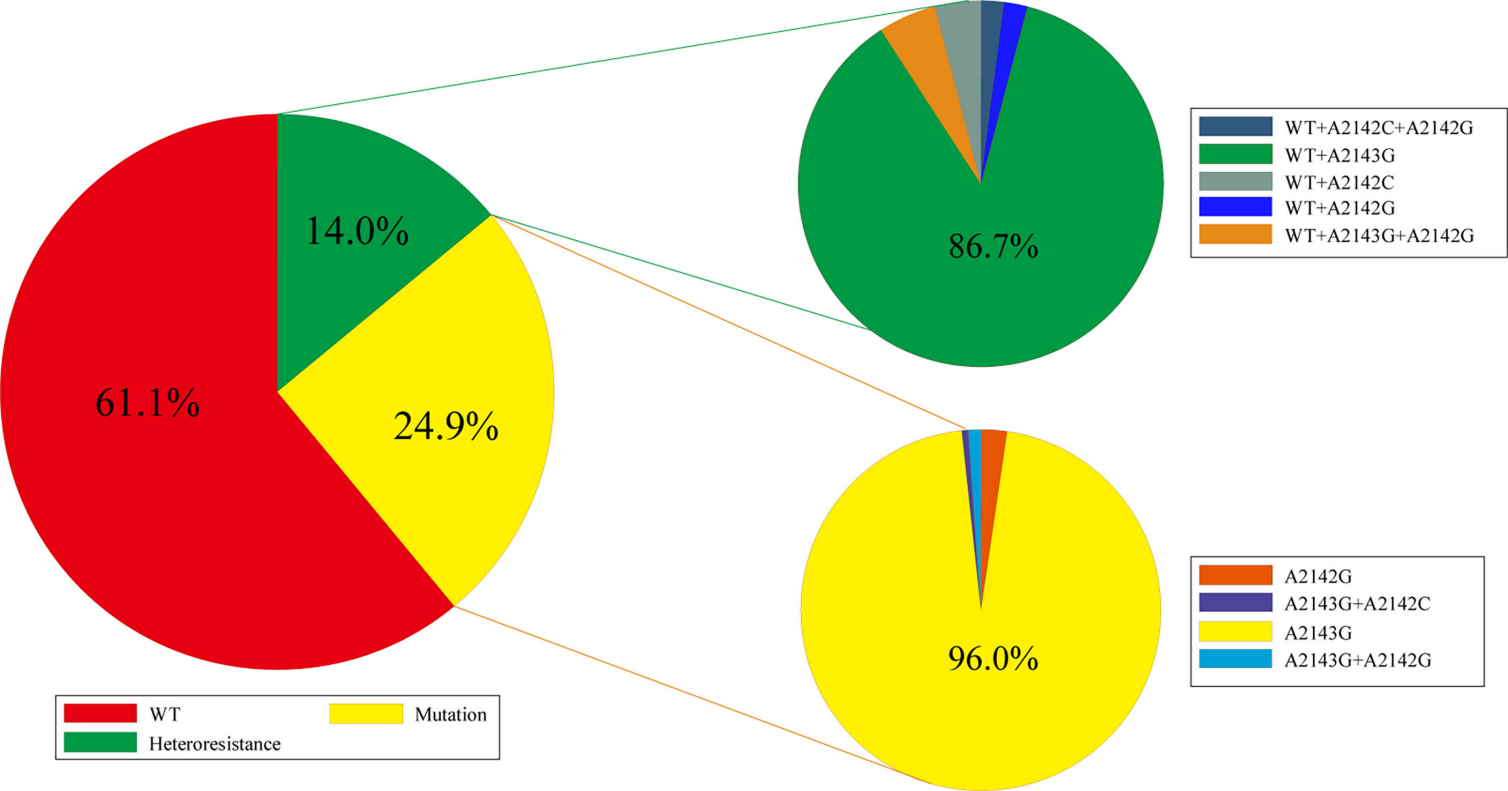
Profile of 23S rRNA genotyping in gastric biopsies (WT, wild type; A2142G, A2143G, and A2142C are point mutations in 23S rRNA gene).

For the 699 biopsies that were positive for 23S rRNA gene detection, 427 (61.1%) showed a WT genotype (2142A, 2143A), 174 (24.9%) showed a 23S rRNA mutation genotype (2142G, 2143G), and 98 (14.0%) showed a heteroresistance genotype (WT+ mutation genotype). For these samples with mutations, the A2143G mutation was predominant, and there were no A2142C mutations alone, but this mutation could exist in combination with A2143G. For these heteroresistance samples, the WT combined with A2143G (86.7%) genotype was also found in most samples, and for these samples, the A2142C mutation was present with the WT genotype or together with A2143G (**Figure 1**).

**Figure 2:**
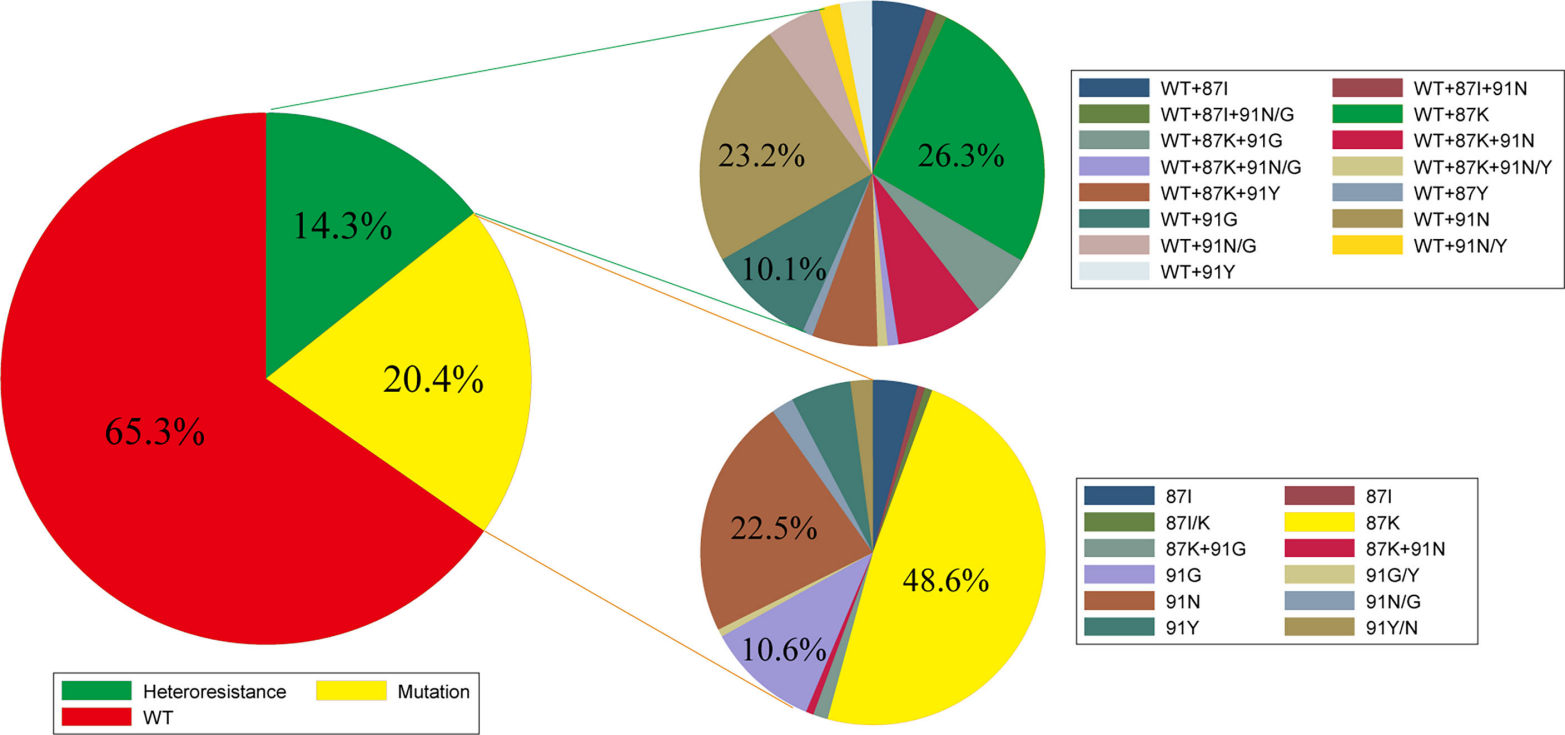
Profile of gyrA genotyping in gastric biopsies (WT, wild type; K, lysine; N, asparagine; Y, tyrosine; G, glycine; I, isoleucine; D, aspartate; 87K, the 87th amino acid was replaced by lysine; 91G, the 91st amino acid was replaced by glycine).

For the 694 biopsies that were positive for gyrA gene detection, 453 (65.3%) showed a WT genotype (87N+91D), 142 (20.4%) showed a gyrA mutation genotype, and 99 (14.3%) showed a heteroresistance genotype. For these samples with mutations, single amino acid mutations, including the mutations 87K (48.6%), 91N (22.5%), and 91G (10.6%), were predominant, and the mutation 87K+91G was found only in a few biopsies. For these heteroresistance samples, the WT combined with 87K (26.3%), 91N (23.2%), or 91G (10.1%) genotype was predominant (**Figure 2**).

We also analyzed the profile of the dual-resistance genotype, and the results showed that 59 (8.6%) samples had genotypes resistant to 23S rRNA and gyrA genes. On the other hand, 22 (3.2%) samples had dual heteroresistance.

### The Consistency Between the Heteroresistance Genotype and Antimicrobial Susceptibility Test Results

A total of 470 *H. pylori* strains were isolated, and AST for CLA and LEV was performed.

When comparing the 23S rRNA genotype with the CLA MIC results, 275 samples had WT 23S rRNA genotypes, while according to the E-test results, the phenotype resistance rate for CLA was 6.2% (17/275). A total of 126 samples had mutation genotypes; according to the E-test results, the phenotype resistance rate was 73.8% (93/126). Sixty-nine of the samples had heteroresistant genotypes; according to the E-test results, the phenotype resistance rate was 36.2% (25/69). To further explore the relationship between the heteroresistance genotype and AST results, we divided these heteroresistance samples into three groups according to the sequencing profile peaks of the mutation position. The results showed that the MUT<WT group had a phenotype resistance rate of approximately 23.5%, the phenotype resistance rate for the MUT≈WT group was 44.4%, and the phenotype resistance rate for the MUT>WT group was 53.8% (**Table 1**). The detailed consistency between the CLA heteroresistance genotype and antimicrobial susceptibility test results is shown in **Supplementary Table 1**. We also noticed that the A2142C mutation was present in a very low peak in the heteroresistant strains.

**Table 1:** Phenotype resistance rates for biopsies with different genotypes.

	WTgenotype	Heteroresistance genotype			Resistance genotype
		MUT<WT	MUT≈WT	MUT>WT	
Phenotype resistance rate for CLA	6.2%	23.5%	44.4%	53.8%	73.8%
Phenotype resistance rate for LEV	15.9%	62.1%	66.7%	78.6%	78.4%

WT, wild type; M<W, the mutation peak was significantly lower than the WT peak; M≈W, the mutation peak was similar to the WT peak; M>W, the mutation peak was significantly higher than the WT peak; CLA, clarithromycin; LEV, levofloxacin.

When comparing the gyrA genotype with the LEV MIC results, 301 samples had the WT gyrA genotype, while according to the E-test results, the phenotype resistance rate was 15.9% (48/301). Ninety-seven samples had a mutation genotype, and according to E-test results, the phenotype resistance rate for LEV was 78.4% (76/97). Seventy-two samples had a heteroresistance genotype, and according to the E-test results, the phenotype resistance rate was 68.1% (49/72). Similarly, we divided these heteroresistant samples into three groups according to the sequencing profile peaks of the mutation position. The results showed that the MUT<WT group had a phenotype resistance rate of approximately 62.1%, the phenotype resistance rate for the MUT≈WT group was 66.7%, and the phenotype resistance rate for the MUT>WT group was 78.6% (**Table 1**). The detailed consistency between the LEV heteroresistance genotype and antimicrobial susceptibility test results is shown in **Supplementary Table 1**.

### The Clinical Relevance of Different Genotypes

We also analyzed the relationship between the different genotypes and clinical disease. According to endoscopy diagnosis data, for the 699 patients who were positive for 23S rRNA gene detection, the WT genotype samples showed a higher peptic ulcer rate (157/427, 36.8%) than the 23S rRNA mutation genotype (43/174, 24.7%) or heteroresistance genotype samples (23/98, 23.5%) (p < 0.05). According to pathologic diagnosis data, the detection rates of severe chronic non-atrophic gastritis or mild intestinal metaplasia among these three groups were similar. The detection rate of moderate intestinal metaplasia was slightly decreased in the WT genotype samples (17/427, 4.0%) compared with the 23S rRNA mutation genotype (13/174, 7.5%) or heteroresistance genotype samples (6/98, 6.1%) (**Table 2**). The detection rates of severe intestinal metaplasia and mild atrophic gastritis were low in all three groups.

**Table 2:** The relationship between different genotype and endoscopy/pathologic diagnoses.

	Genotype	PU	SC-NAG	Mi-IM	Mo-IM	S-IM	M-AG
**CLA**	WT	157 (36.8%)	51 (11.9%)	92 (21.5%)	17 (4.0%)	1 (0.2%)	3 (0.7%)
	Mutation	43 (24.7%)	12 (6.9%)	41 (23.6%)	13 (7.5%)	1 (0.6%)	1 (0.6%)
	Heteroresistance	23 (23.5%)	8 (8.2%)	20 (20.4%)	6 (6.1%)	0 (0.0%)	1 (1.0%)
**LEV**	WT	172 (38.0%)	47 (10.4%)	104 (23.0%)	20 (4.4%)	1 (0.2%)	2 (0.4%)
	Mutation	29 (20.4%)	15 (10.6%)	26 (18.3%)	9 (6.3%)	1 (0.7%)	2 (1.4%)
	Heteroresistance	23 (23.2%)	10 (10.1%)	24 (24.2%)	7 (7.1%)	0 (0.0%)	1 (1.0%)

PU, peptic ulcer; SC-NAG, severe chronic non-atrophic gastritis; Mi-IM, mild intestinal metaplasia; Mo-IM, moderate intestinal metaplasia; S-IM, severe intestinal metaplasia; M-AG, mild atrophic gastritis; WT, wild type; CLA, clarithromycin; LEV, levofloxacin.

According to the endoscopy diagnosis data, for the 694 patients who were positive for gyrA gene detection, the WT genotype samples showed a higher peptic ulcer rate (172/453, 38.0%) than the gyrA mutation genotype (29/142, 20.4%) or heteroresistance genotype samples (23/99, 23.2%) (p < 0.05). According to the pathologic diagnosis data, the detection rates of severe chronic non-atrophic gastritis or mild intestinal metaplasia among these three groups were similar. The detection rate of moderate intestinal metaplasia was seemingly lower in the WT genotype samples (20/453, 4.4%) than in the gyrA mutation genotype (26/142, 6.3%) or heteroresistance genotype samples (7/99, 7.1%) (**Table 2**). The detection rates of severe intestinal metaplasia and mild atrophic gastritis were low in all three groups.

## Discussion

The decrease of eradication rates of anti-*H. pylori* treatment has been a research hotspot in recent decades. Antimicrobial resistance has been thought to be the main reason for this situation. The eradication rate with bismuth-quadruple therapy was significantly higher than that with standard triple therapy in the group with the A2143G mutation or with the double mutation (Kim et al., 2021). In Europe, the resistance rate for CLA and LEV was approximately 21.4% and 15.8%, respectively (Megraud et al., 2021). In the United States, the prevalence of CLA resistance was 32.3%. In China, the primary antibiotic resistance rates of *H. pylori* were 37.00% for CLA and 34.21% for LEV (Zhong et al., 2021). If heteroresistance cases are considered, the resistance rate for many antibiotics would be higher (Pereira et al., 2021; Wang et al., 2021).

Heterogeneous antibiotic resistance was first described in 1947 for the gram-negative bacterium *Haemophilus influenzae*. In 1999, Marais et al. suggested that the use of PCR/DNA enzyme immunoassays allows the detection of multiple genotypes corresponding to either heterogeneous genotypes or mixed infections (Marais et al., 1999). Currently, it has been proven that heteroresistant genotypes appear not only in phenotype-resistant samples but also in phenotype-susceptible samples. As our work shows, *H. pylori* heteroresistance for CLA and LEV is common, and the most common heteroresistance genotype for CLA was WT combined with A2143G. The most common heteroresistance genotype for LEV was WT combined with 87K. Although mutations such as A2142C are the most efficient mutations for CLA resistance (Wang et al., 2020), the frequency of this mutation was very low; in heteroresistant samples, the peak of this mutation was very low, which suggests that this mutation may have no growth advantage in CLA-free conditions. As some research suggests, the pathogen may take toward complete resistance or, sometimes, toward the loss of a resistance factor that exerts an excessive fitness cost in the absence of antibiotic pressure (Andersson et al., 2019).

Susceptible populations would act as the majority population of heteroresistance cases. We explored whether heteroresistance samples show susceptible phenotype in most cases. We compared the consistency of heteroresistant genotypes with phenotype-susceptible data. Our results show that most CLA heteroresistance cases showed a susceptible phenotype, and many LEV heteroresistance cases showed a resistance phenotype, which suggests that when we use a traditional CLA-based method for tailored anti-*H. pylori* treatment, there is a chance that applying CLA-containing treatment for heteroresistance cases may result in eradication failure. This approach is currently the most widely used method for tailored treatment (Cha et al., 2021; Choe et al., 2021; Perkovic et al., 2021). And for LEV heteroresistance cases, resistance phenotype may be a relatively stable state without too much fitness cost. And there is also some chance of applying LEV-containing treatment for heteroresistance cases.

We were also interested in the clinical relevance of heteroresistance cases. Unfortunately, due to the high resistance rate of CLA and LEV (Liu et al., 2018), treatments containing CLA or LEV can no longer act as first-line anti-*H. pylori* treatments in our hospital, so we could not evaluate the relationship between heteroresistance and eradication failure. Instead, we collected data from clinical and pathologic diagnosis data. Interestingly, we found that cases with WT genotypes showed a higher peptic ulcer rate than cases with mutation and heteroresistance genotypes. On the other hand, the detection rate of moderate intestinal metaplasia indicates a slight decrease in cases with the WT genotype. This result suggests that the existence of heteroresistant strains may be asymptomatic in patients but induce more precancerous lesions. Though we do not have data for heteroresistance and eradication failure, the evidence that antibiotic heteroresistance is responsible for treatment failure in clinical settings is increasing. Thus, detection and characterization of heteroresistance would be important for appropriate therapeutic guidance to treat bacterial infections.

## Conclusions

Heteroresistance genotypes for CLA and LEV were not rare in *H. pylori*, most heteroresistance genotype cases showed susceptible phenotypes for CLA and resistance phenotypes for LEV, and patients infected with heteroresistance genotype strains showed a lower peptic ulcer detection rate than those infected with the WT strain.

## Data Availability Statement

The original contributions presented in the study are included in the article/**Supplementary Material**. Further inquiries can be directed to the corresponding authors. The data presented in the study are deposited in the NCBI BankIt repository, accession number Banklt2529337:OL854226 - OL855603.

## Ethics Statement

The research protocol was approved by the Ethics Committee of the First Affiliated Hospital of Nanchang University (IRB 2018-116). The patients/participants provided their written informed consent to participate in this study.

## Author Contributions

Y-hW and X-lG: acquisition of the data, analysis and interpretation of the data, drafting of the manuscript, and statistical analysis. D-wL and RZ: acquisition of the data, and analysis and interpretation of the data. L-fZ and X-yS: critical revision of the manuscript for important intellectual content. YX and D-sL: study concept and design, obtained funding, and critical revision of the manuscript for important intellectual content. Y-hW and X-lG contributed equally to this work. All authors read and approved the final manuscript.

## Funding

Funding was provided by grants from the National Natural Science Foundation of China (81970502, 81460115, and 82060109), the National Key Research and Development Program of China (2016YFC1302201), the Key Research and Development Program of Jiangxi Province Department of Education (20203BBG73051), and the Science and Technology Projects of Jiangxi Province (GJJ180047) and Scientific Research Project of Jiangxi Drug Administration (2020JS22).

## Conflict of Interest

The authors declare that the research was conducted in the absence of any commercial or financial relationships that could be construed as a potential conflict of interest.

## Publisher’s Note

All claims expressed in this article are solely those of the authors and do not necessarily represent those of their affiliated organizations, or those of the publisher, the editors and the reviewers. Any product that may be evaluated in this article, or claim that may be made by its manufacturer, is not guaranteed or endorsed by the publisher.
