# Supplementary material for: Characteristics of Helicobacter pylori Heteroresistance in Gastric Biopsies and Its Clinical Relevance
Source: Front Cell Infect Microbiol. 2022 Feb 4;11:819506. doi: 10.3389/fcimb.2021.819506 (PMC8855363; doi:10.3389/fcimb.2021.819506)
Supplement: Supplementary file 1 [file Table_1.docx]

Supple Table 1.

|  | MUT>WT | | MUT≈WT | | MUT<WT | |
| --- | --- | --- | --- | --- | --- | --- |
|  | **R** | **S** | **R** | **S** | **R** | **S** |
| WT+A2143G | 13 | 11 | 3 | 4 | 6 | 21 |
| WT+A2143G+A2142G | 0 | 0 | 1 | 1 | 0 | 2 |
| WT+A2142C | 0 | 0 | 0 | 0 | 1 | 3 |
| WT+A2142C+A2142G | 1 | 0 | 0 | 0 | 1 | 0 |
| WT+A2142C+A2143G | 0 | 1 | 0 | 0 | 0 | 0 |
|  |  |  |  |  |  |  |

| WT+87K | 4 | 2 | 1 | 2 | 6 | 4 |
| --- | --- | --- | --- | --- | --- | --- |
| WT+91N | 5 | 2 | 0 | 2 | 4 | 3 |
| WT+91G/D | 3 | 0 | 1 | 0 | 1 | 2 |
| WT+87K+91Y | 3 | 1 | 2 | 0 | 0 | 0 |
| WT+87K+91N | 2 | 0 | 1 | 0 | 3 | 0 |
| WT+91N/G | 2 | 1 | 0 | 0 | 1 | 1 |
| WT+87K+91G | 1 | 0 | 3 | 0 | 0 | 0 |
| WT+87I | 0 | 0 | 1 | 0 | 1 | 1 |
| WT+91N/Y | 2 | 0 | 0 | 0 | 0 | 0 |
| WT+87K+91N/Y | 0 | 0 | 1 | 0 | 0 | 0 |
| WT+91Y | 0 | 0 | 0 | 1 | 0 | 0 |
| WT+87Y | 0 | 0 | 0 | 0 | 1 | 0 |
| WT+87I+91N/G | 0 | 0 | 0 | 0 | 1 | 0 |

(M<W: the mutation peak was significant lower than the WT peak, M≈W: the mutation peak was similar to the WT peak, M>W: the mutation peak was significant higher than the WT peak, R: resistant, S: susceptible; WT: wild type, K: lysine, N: asparagine, Y: tyrosine, G: glycine, I: isoleucine, D: aspartate).
