# Supplementary figures and images for: Characteristics of Helicobacter pylori Heteroresistance in Gastric Biopsies and Its Clinical Relevance
Source: Front Cell Infect Microbiol. 2022 Feb 4;11:819506. doi: 10.3389/fcimb.2021.819506 (PMC8855363; doi:10.3389/fcimb.2021.819506)

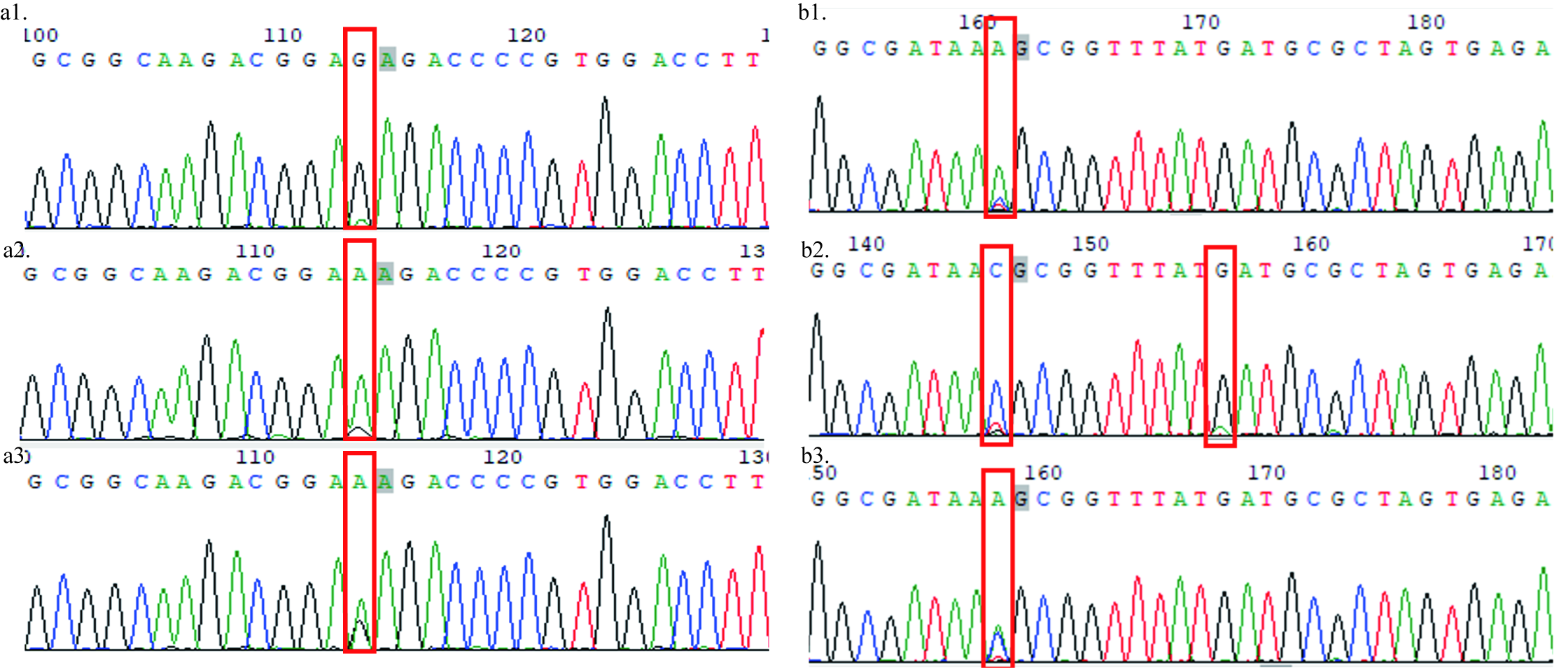

Supplement: Supplementary file 2 [file Image_1.tif]
